# P-157. Molecular epidemiology of carbapenemase genes among carbapenem-resistant *Klebsiella pneumoniae* (CR-*Kp*) from the Philippines: implications for surveillance and treatment

**DOI:** 10.1093/ofid/ofae631.362

**Published:** 2025-01-29

**Authors:** Edsel Maurice Salvana, Jonnel B Poblete, Christian Francisco, Stessi Geganzo, Vivienne Luzentales, Ma Grace Hernaez, Angelo dela Tonga, Federico Perez, David Paterson, Robert A Bonomo

**Affiliations:** Institute of Molecular Biology and Biotechnology, National Institutes of Health, University of the Philippines, Manila, National Capital Region, Philippines; Philippine General Hospital, University of the Philippines, Manila, Manila, National Capital Region, Philippines; National Institutes of Health, University of the Philippines Manila, Manila, National Capital Region, Philippines; National Institutes of Health, University of the Philippines Manila, Manila, National Capital Region, Philippines; National Institutes of Health, University of the Philippines Manila, Manila, National Capital Region, Philippines; National Institutes of Health, University of the Philippines Manila, Manila, National Capital Region, Philippines; National Institutes of Health, University of the Philippines Manila, Manila, National Capital Region, Philippines; Case Western Reserve University, Cleveland, OH; The University of Queensland Centre for Clinical Research, Brisbane, Queensland, Australia; Case Western Reserve University/ Louis Stokes Cleveland VA Medical Center, Cleveland, OH

## Abstract

**Background:**

In the Philippines, the proportion of carbapenem-resistant *Klebsiella pneumoniae* (CR-*Kp*) has doubled in the past decade and now exceeds 15%. However, the prevalence of different carbapenemase genes remains unknown. Unlike the United States and China where *bla*_KPC_ predominates, *bla*_OXA-48_-like serine carbapenemases and metallo-β-lactamases (MBL) *bla*_NDM_, -_IMP_, and -_VIM_ frequently occur among CR-*Kp* in the Asia-Pacific region. In order to better inform empiric treatment strategies and assist with infection prevention, we investigated the carbapenemases present in CR-*Kp* from the Philippine General Hospital (PGH), a 1,500-bed tertiary care national referral center in Manila, Philippines.

**Table 1.**
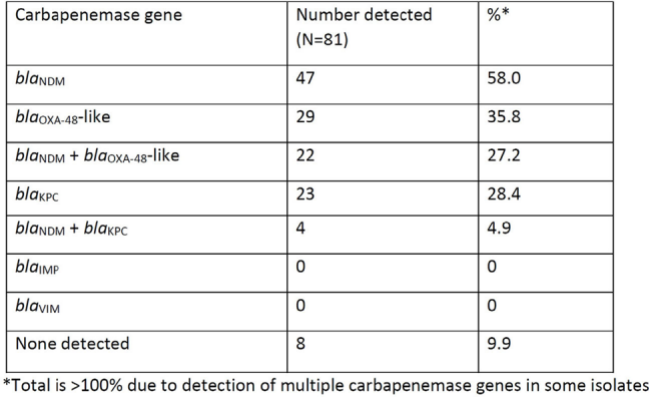
Carbapenemase genes detected with Xpert® Carba-R among carbapenem-resistant Klebsiella pneumoniae isolates from the Philippine General Hospital in Manila, Philippines.

**Methods:**

CR-*Kp* from clinical samples were identified in the clinical microbiology laboratory at PGH using the VITEK® 2 platform (bioMerieux, USA). These isolates were collected and underwent rapid molecular testing with Xpert® Carba-R (Cepheid, USA) at the central laboratory of the Philippine National Institutes of Health. Molecular test results were typically available within 24 hours from the time of identification. These results were relayed to the treating physicians to guide clinical decision-making.

**Results:**

A total of 81 CR-*Kp* isolates were collected from February to May 2024. Isolates were obtained from respiratory (n=44, 54.3%), blood (n=24, 29.6%), urine (n=9, 11.1%), and tissue/wound (n=4, 4.9%) samples. Table 1 summarizes the carbapenemase genes detected with Xpert® Carba-R among CR-*Kp* isolates from PGH, including multiple genes present in some samples.

**Conclusion:**

Among CR-*Kp* from PGH, *bla*_NDM_ was the predominant carbapenemase, followed by *bla*_OXA-48_-like and *bla*_KPC_ genes. A third of the isolates co-harbored *bla*_NDM_ and a serine carbapenemase, usually *bla*_OXA-48_-like. These findings suggest that there is molecular heterogeneity and likely plasmid spread among CR-*Kp* in the Philippines. These findings also underscore the importance of molecular surveillance programs that can rapidly characterize resistant strains, highlight the emergence of novel genotypes, and inform empiric and definitive therapy for CR-*Kp*.

**Disclosures:**

**Federico Perez, MD, MS**, Merck: Grant/Research Support **David Paterson, Infectious Diseases Physician**, AMR Action Fund: Board Member|Aurobac: Advisor/Consultant|bioMerieux: Grant/Research Support|bioMerieux: Honoraria|CARB-X: Advisor/Consultant|Entasis: Expert Testimony|Menarini: Honoraria|Merck: Grant/Research Support|Pfizer: Advisor/Consultant|Pfizer: Grant/Research Support|Shionogi: Grant/Research Support|Shionogi: Honoraria

